# Sarcomatous Tumors in the Orbit

**Published:** 1880-04

**Authors:** Lucien Howe


### CLINICAL REPORTS

### SARCOMATOUS TUMORS IN THE ORBIT.

BY LUCIEN HOWE, M. D.

CASE No. I. E. M., was a woman of forty, who usually enjoyed excellent health. About 1873 she noticed a "small black speck" on the lower and inner portion of the conjunctiva of the right eye. This gradually increased in size till on the 3d of April, 1877, she presented herself at the Buffalo Eye and Ear Infirmary. The condition on that day was recorded substantially as follows:

Lower lid pushed down and outwards half an inch. Between its internal two-thirds, and the globe of the eye, there exists a tumor which measures 1⅛ inch horizontally, ⅝ inch vertically, and projects ½ inch beyond the edge of the lower lid. It is dark colored, vascular, but not compressible; movable, not tender. The globe of the eye is pushed up and backwards, and partly hidden by the growth and its movements in different directions considerably impeded. Moreover, on the internal and inferior quadrant of the cornea, there is a spot infiltrated with pus, and the vision of the eye markedly impaired—more exactly $V=\frac{20}{100}$. Pain is not a constant symptom, but frequently felt in the eye and its vicinity.

On account of the interesting features of this case, the patient was exhibited at a meeting of the Buffalo Medical Association, in April of that year, and various opinions were expressed as to the prognosis. One gentleman, of wide surgical experience had even been led to think that the removal of growths decidedly "malignant" would give in general only temporary relief, and was therefore to be discouraged.

Operative interference was not delayed, however, and on the 7th of April, chloroform was administered, and assisted by Doctors Abbott and McNiel, I attempted the removal of the growth. In its deeper portions, however, it was found to have invaded the globe of the eye to such an extent that it was necessary to remove that also. Especial care was taken to extirpate every trace of the growth, and the incisions were extended on every side into tissue, which seemed to be healthy. A light bandage was applied, the patient rode home in the horse-cars, and apparently suffered but little inconvenience from the procedure. By the third day the reparative process was well established in the outer portion of the wound, while the parts formerly contiguous to the tumor were much swollen and covered with a thick layer of pus.

A subsequent examination showed that a minute spot, similar in appearance to the growth, still remained in the orbit and suspecting that in spite of all precautions, a portion had been left, still further removal of the tissue was deemed advisable. Chloroform was again administered therefore, and the contents of the orbit in its lower and inner angle entirely extirpated, the incisions in this locality being carried well down on to the bone. Healthy granulations appeared soon afterwards, the wound healed rapidly and no further annoyance has since been experienced. A microscopic examination of the growth showed it to be a melanotic sarcoma of the spindle-celled variety.

Case No. II. J. B. C., a farmer, 54 years old, from the town of Colden, in this state, consulted me on the 5th of last December, concerning a growth at the inner angle of the right eye. Five years before he noticed a small projection in the conjunctiva a little external to the caruncle. This gradually increased in size for two years, when it was partially "eaten out by a plaster" applied by an irregular practitioner. As a result of that attempt, extensive cicatrices were formed which produced marked ectropium of the lower lid and also drew the upper one down far over the globe. This condition of the lids persisted when the patient consulted me, and the tumor in the meanwhile had attained the size of a small walnut. It occupied the entire inner angle of the eye, being attached to the inner portion of the lids above and below. It had pushed the eye-ball outwards extending over nearly half of its internal surface and was firmly attached to the tissues about the lachrymal bone. It was of a bright red color, quite vascular, not compressible nor tender and movable only at its outer portion. Pain was experienced only occasionally but was of a sharp, lacerating character. Under such circumstances there seemed to be no question as to the advisability of operative interference. Accordingly, ether was administered on the 13th of December, and the growth completely removed. There were found to be no attachments to the globe of the eye, and it was therefore allowed to remain, but all the tissue in the vicinity of the base of the tumor was thoroughly extirpated.

One incision was carried obliquely upwards along the superior margin as far in as the orbital ridge and lachrymal bone; another extended obliquely downward along its inferior margin quite to the nasal process of the superior maxillary bone, and everything included in this triangle, with the exception of the lids, was carefully dissected out. The wound healed rapidly, and thus far there is not the least symptom of any recurrence of the disease. The microscopic appearances of the tumors were such as characterize a sarcoma, the round cells being thickly strewn through a connective tissue stroma. Its general structure was like that of the alveolar, round-celled sarcoma, figured by Rindfleisch,* provided no pigment were visible.

These cases appear to be of interest; 1st, as examples of a rare form of disease; 2d, in the favorable result thus far of the operation; 3d, as furnishing some indications as to the extent to which the tissue adjacent to such tumors shall be sacrificed.

In regard to the frequency of these growths in the orbit, an approximation was attained, by examining the statistics found in the reports of ophthalmic hospitals and infirmaries, in which an aggregate of over thirty-eight thousand patients had been treated. This showed that only one such case occurred, on the average, in over four thousand, one hundred and twenty others of diseases of the eye.

* Lehrbuch der Pathologische Gewebelehre—Fig. 45.

Again, the sarcomata are specially "malignant," and almost invariably terminate fatally. This is particularly true of the new formations of which the first case is illustrative. An eminent writer expresses the views of many others in saying "*these melanotic tumors are amongst the most malignant of the sarcomatous growths." As further showing the dreadful results of this class of diseases, the writer would refer to the interesting cases reported by Doctors †Dyer ‡Hag and ||Higgins. All the cases which I have thus far found on record, and in which the conditions were similar to No. 1 here given, have proved fatal within two years and eight months.

Finally, the result in the two cases here described, would seem to indidate that an early operation, with very free extirpation of the adjacent tissue would, in some instances, arrest the progress of the malady. The fact that the growth recurred in the orbit of the woman, after *all* those portions had been thoroughly removed, which could be recognized by the naked eye as in the least abnormal, and the fact that the wound healed so completely after the second operation, would appear to show that in the extirpation of malignant tumors, wherever found, we should not hesitate to include the adjacent tissue to as great an extent as it can be spared. Neither sight nor touch furnish us any positive data as to the extent to which the contiguous portions are involved, and no attempt at operation should be made, unless we remove every perceptible trace of the growth and as much more as can, with propriety, be sacrificed.

---

* Pathology and Morbid Anatomy—Green—English Edition, p. 120.
† Trans. American Ophthalmological Society, vol. ii, page 538.
‡ Trans. International Ophthalmological Congress.
|| British Med. Journal, Dec. 8, 1877.

